# Renal Pelviceal Keratinizing Squamous Metaplasia with Sparing of Pyramidal Zones

**DOI:** 10.1155/2012/242780

**Published:** 2012-01-26

**Authors:** Richard H. Siderits, Jared Fingerman, Anup Hazra, Cheryl Rimmer, Marc Colaco, Nagy Mikhail, Cristian Ardeleanu, Peter M. Mazari

**Affiliations:** ^1^Department of Pathology, Robert Wood Johnson University Hospital, Hamilton, NJ 08690-3599, USA; ^2^Lawrenceville Urology, Lawrenceville, NJ 08648, USA; ^3^Robert Wood Johnson University Medical School, New Brunswick, NJ 08903, USA; ^4^Southern Ocean County Hospital Pathology, Robert Wood Johnson University Hospital, New Brunswick, NJ 08901, USA

## Abstract

Metaplastic changes in the urothelium of the upper urinary tract are relatively infrequent. Metaplasia may present as either squamous or less often glandular differentiation. The process may be associated with chronic inflammation or associated chronic infections. There may be malignant transformation to either squamous cell carcinoma or adenocarcinoma. The demarcation of the metaplastic process in the minor calyces has not been well documented to date. We report the case of a 74-year-old female patient who presented with a history of chronic renal disease and acute pyohydronephrosis. The patient underwent a nephroureterectomy which revealed keratinizing desquamative squamous metaplasia throughout the renal pelvis and upper urinary tract with abrupt termination of metaplasia at the junction of the renal pelvis and the minor calyx (pyramidal zone). Immunohistochemical evaluation documents metaplastic urothelium stained positive for CK5, before converting sharply to simple cuboidal epithelium in the minor calyx (pyramidal zones) which stained positive CK7. At the junction of the metaplastic components and low cuboidal lined minor calyceal surfaces, the underlying stroma showed loss of ureteral muscularis mucosa with transition to renal parenchymal type stroma. We believe that this observation is unique and potentially relevant to the etiology and pathophysiology of pelviceal metaplasia.

## 1. Introduction

Keratinizing desquamative squamous metaplasia (KDSM) is a condition in which urothelium of the urinary tract is replaced with keratinized squamous epithelial cells. While such metaplasia within the lower urinary tract is not uncommon, KDSM of the upper urinary tract and renal pelvis is rarer [1]. Originally referred to as renal leukoplakia or cholesteatoma, KDSM has an uncertain etiology. The condition has been associated with chronic irritant exposure or infection as may be seen with xanthogranulomatous pyelonephritis, nephrolithiasis, syphilis, tuberculosis, and cigarette smoking [2, 3]. It has also been documented in young children, suggesting a possible genetic component [4]. KDSM was believed to have been a precancerous process with malignant transformation approaching 8%–12% of cases [5]. This proportion would be similar for instances of intestinal type glandular metaplasia involving the upper urinary tract. There are however too few documented cases to generate statistically significant data [6]. For this reason, the notion of KDSM representing a premalignancy has been challenged and some researchers suggest that endoscopic management and close followup should replace radical nephroureterectomy as the preferred treatment [7–9]. Nevertheless, most cases of KDSM result in nephroureterectomy, more than likely because the condition is difficult to distinguish from other malignant neoplastic processes (including transitional cell carcinoma, Wilms tumor, and intrarenal teratoma) preoperatively [10, 11]. 

We report a case of KDSM of the renal pelvis with the novel observation of universal sparing of the minor calices and renal papillae in a 74-year-old woman with a five-year history of ureteral obstruction and chronic pyelonephritis. The squamous metaplasia in the hydronephrotic kidney appeared to end abruptly at the junction of the renal pelvis and the minor calyceal system. To the best of our knowledge, this observation has not been reported in previous cases of KDSM of the upper urinary tract system. We postulate that the abrupt transition from metaplastic squamous to low cuboidal epithelium may reflect a differential metaplastic response and posit a role for consequent stromal induction for the metaplastic process.

## 2. Case Report

The patient was a 74-year-old female presenting with a clinical history significant for chronic kidney disease with repeated unilateral renal obstruction and chronic hydronephrosis. The patient had undergone ureteral stenting every six months for the previous five years. The patient was documented to have chronic infection with Extended Spectrum Beta Lactamase producing *klebsiella pneumoniae*. At presentation there was an obstruction at the level of the renal pelvis resulting in pyohydronephrosis. Ureteroscopy revealed a cobblestone appearance. The bladder appeared unremarkable, and the opposite kidney showed compensatory changes but was otherwise unremarkable. Past medical history was significant for diabetes and controlled hypertension. There was no documented history of urinary reflux, exstrophy, or paralysis.

**Figure 1:**
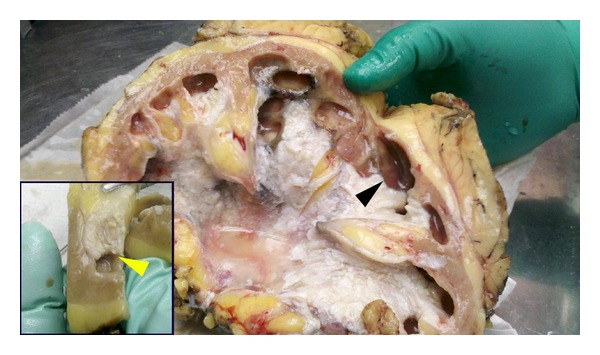
Macroscopic findings showing squamous metaplasia of renal pelvis and abrupt sparing of pyramidal zones. Insert lower left shows metaplastic process stops at transition zone.

 A right nephroureterectomy was performed. The resulting surgical specimen consisted of a macroscopically recognizable kidney weighing 240 grams and measuring 18.0 × 12.0 × 5.8 cm, devoid of perirenal fat. The specimen was bisected, revealing dilated renal papillae and pelviceal components. The pelvis contained caseous, foul-smelling material with caseous appearance that was reminiscent of an epidermal inclusion cyst. The surface of the renal pelvis showed a pearly gray-to-silver sheen with foci of black discoloration (Figure 1). Interestingly the dilated hydronephrotic papillae showed no evidence of discoloration or involvement by the pathological process.

**Figure 2:**
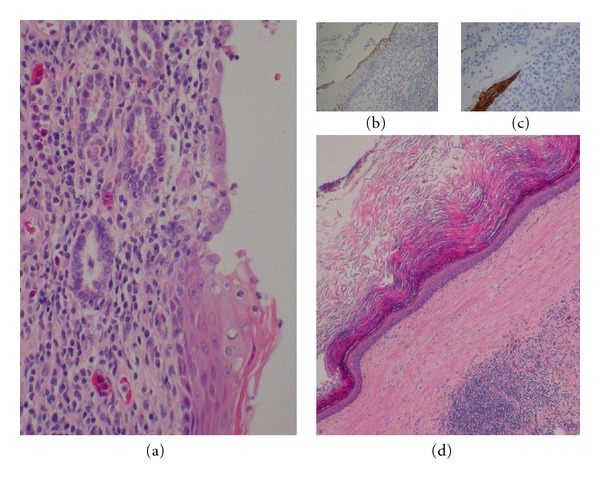
Histologic findings at 40x (a) show H&E stained transition zone between renal parenchymal stroma (top) and renal pelviceal stroma (bottom); (b) shows metaplastic epithelial component is negative for CK5+; (c) shows CD7+ positive staining of metaplastic component; (d) shows extensive desquamative squamous metaplasia over densely collagenized pelviceal stroma.

Sections of the renal pelvis showed desquamative squamous metaplasia in the absence of adnexal structures or tissue analogs which might have been suggestive of an intrarenal teratoma. The squamous metaplastic process appeared to end abruptly at the juncture of the pelvis and minor calyceal system (insert, Figure 1). Histologic features are seen in Figure 2(a) (H&E stain). Metaplastic changes in the urothelium of the renal pelvis stained CK5+/CK7− (Figure 2(b) insert), with abrupt transformation to simple cuboidal epithelium (lining the minor calyx) which stained CK5−/CK7+ (Figure 2(c) insert); CK7 would have been the usual cytokeratin found in the collecting ducts. Additionally, at the juxtaposition of these two surfaces, we see changes in the underlying stroma with the transitional loss of the ureteral muscularis mucosa with transition to underlying renal parenchyma in the pyramidal zone of the minor calyx (Figure 2(d) insert).

## 3. Discussion

This report describes a case of Keratinizing Desquamative Squamous Metaplasia (KDSM) extending from the upper ureter and renal pelvis to the junction of the major and minor calyces with abrupt transformation to low cuboidal urothelium at the margin of the minor calyces. This process has arisen within a background of long-standing chronic inflammation, *klebsiella* infection, and subsequent hydronephrosis. The most striking feature was the abrupt transition from metaplastic squamous epithelium to low cuboidal urothelium in the minor calyces around the renal pyramids. To our knowledge, this is a novel observation and has not been previously reported in the medical literature.

The clinical import of this observation suggests that even left untreated KDSM would not extend into the kidney parenchyma and would likely preserve the ability of the nephron to drain through the renal pelvis. This also supports the current conservative trend of ureteroscopic management. Since ureteral obstruction by desquamated keratinous material appears to be the etiology of hydronephrosis and/or pyelonephritis, alleviation of the obstruction should preserve kidney function.

The immunohistological findings further accentuate the striking morphological differences between the metastatic and normal epithelia. The superficial metaplastic squamous epithelium stains positives for cytokeratin 5 (CK5), a filament typically found in all layers of squamous epithelium but only the basal and intermediate layers of urothelium [12]. Meanwhile, the unaffected tissues stain positively for cytokeratin 7 (CK7) (a filament found in urothelium but absent from squamous epithelium), and both CK5 and CK7 staining end abruptly at the edge of their respective epithelia. While these changes are clearly abnormal, KDSM as it presents in this case should not inhibit the functioning of the renal pelvis as its relatively large lumen makes it unlikely that it will become occluded by desquamated keratin except in severe cases as is seen in renal cholesteatoma [13]. Conversely the narrower passages of the minor calyces could more easily be obstructed and thus would seem to be evolutionarily disadvantageous to undergo a response like KDSM in the collecting ducts and more proximal structures of the kidney. The nature of the changes does, however, pose several questions regarding the genesis of KDSM and its pathophysiology.

 Metaplasia appears to be a protective adaptation in response to a chronic insult, converting the transitional epithelium to a more robust keratinized squamous epithelium, thereby mitigating damage to underlying stroma. Animal studies have shown stromal-epithelial tissue interactions to induce epithelial differentiation during organogenesis [14], and thus it stands to reason that stromal damage may be involved in the induction of epithelial metaplasia. This hypothesis is supported by our current findings as evidenced by the transitional change of stroma that occurs at the junction between the metaplastic squamous epithelium and the normal urothelium. Like the epithelium, the stroma types show a sharp demarcation between types and the stromal border coincides with the epithelial border.

 There is still debate as to whether KDSM is precancerous or more of a benign process. Some cases of KDSM have been seen in association with squamous cell carcinoma but in other cases long-term observation has not revealed cancerous transformation [5, 8]. KDSM may provide a useful model with which to gain a better understanding of the pathophysiology of metaplasia, especially the role and influence of the underlying stroma for induction of metaplasia and its association with or progression to cancer. We are not aware of a specific reference in the embryologic literature to this apparent juncture and believe that the underlying renal stroma acts as an inducer for the overlying urothelial-derived surface.
